# Sustained mitigation of ST-segment elevation in a patient with Brugada syndrome type 1 during sevoflurane and remifentanil anesthesia: a case report

**DOI:** 10.1186/s40981-024-00702-7

**Published:** 2024-03-12

**Authors:** Kurumi Saito, Hitoshi Yoshida, Kazuyoshi Hirota

**Affiliations:** 1 Department of Anesthesiology, Hirosaki General Medical Center, 1 Tomino-Cho, Hirosaki, 036-8174 Japan; 2 Department of Anesthesiology, Hirosaki University Graduate School of Medicine, 5 Zaifu-Cho, Hirosaki, 036-8562 Japan

**Keywords:** Brugada syndrome, Sevoflurane, ST-segment

## Abstract

**Background:**

During general anesthesia, patients with Brugada syndrome are at risk of malignant arrhythmias following worsened ST-segment elevation, potentially leading to sudden cardiac death. The protocol for safe anesthetic management of patients with Brugada syndrome has not yet been established.

**Case presentation:**

A 63-year-old man, diagnosed with a spontaneous Brugada type 1 pattern, was scheduled for a pleural biopsy using video-assisted thoracoscopic surgery under general anesthesia. We planned general anesthesia using volatile induction and maintenance anesthesia with sevoflurane and remifentanil. We monitored ST-segment morphology and observed sustained mitigation of ST-segment elevation throughout general anesthesia.

**Conclusion:**

The present case may indicate that safe anesthetic management of patients with Brugada syndrome depends on whether the anesthetics used can reduce ST-segment elevation.

## Introduction

Brugada syndrome is an inherited disease characterized by right bundle branch block and ST-segment elevation in the right precordial leads of the electrocardiogram (ECG) caused by ion channel disorders of the cardiac conduction system [1, 2]. In such patients, exacerbation of ST-segment elevation due to the heterogeneity of repolarization between the epicardium and endocardium in the right ventricular outflow tract increases the risk of ventricular tachycardia/fibrillation (VT/VF), potentially leading to sudden cardiac death [3, 4]. In some cases, the ST-segment elevation increases just prior to the onset of polymorphic VT/VF [5].

In the perioperative periods, certain drugs and conditions have been identified as triggering worsened ST-segment elevation and critical arrhythmias in Brugada syndrome patients, most notably ketamine, local anesthetics, hyperthermia, and electrolyte anomalies [6]. Sevoflurane and propofol are widely used general anesthetics in current anesthetic practice, but their use with caution in patients with Brugada syndrome is still recommended [6]. On the other hand, some studies revealed that induction with propofol bolus and volatile-based anesthesia caused reduction or no exacerbation of ST-segment elevation without VT/VF [7–9]. Although there may exist safe methods of anesthetic management in patients with Brugada syndrome that attenuate ST-segment elevation, to our knowledge there are no reports of ST-segment morphology throughout general anesthesia for major surgery in such patients. We report a case of sustained mitigation of ST-segment elevation during anesthesia with only sevoflurane and remifentanil in a patient with Brugada syndrome. Written informed consent was obtained from the patient for the publication of this case report. This manuscript adheres to the applicable EQUATOR guidelines.

### Case presentation

**Fig. 1:**
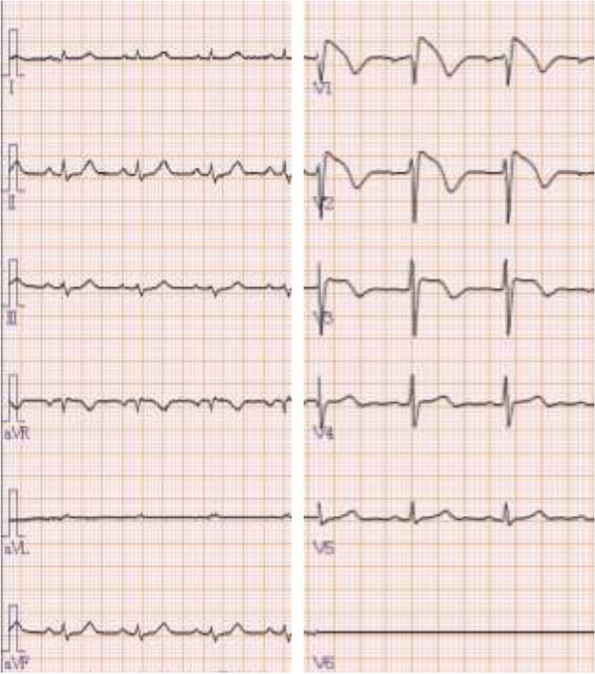
Preoperative 12-lead electrocardiogram showing coved-type ST-segment elevation and J waves amplitude > 0.2 mV in the right chest leads

A 63-year-old man [height 162 cm; weight 53 kg] presented to his family doctor with persistent cough and dyspnea. Chest computed tomography showed multiple pleural masses and left pleural effusion, for which a chest drain was inserted. He was scheduled for a pleural biopsy using video-assisted thoracoscopic surgery (VATS) under general anesthesia to establish the definitive diagnosis of malignant pleural mesothelioma. His preoperative electrocardiogram (ECG) showed coved-type ST-segment elevation and J-wave amplitude > 0.2 mV in the right chest leads (Fig. 1). He was diagnosed with a spontaneous Brugada type 1 pattern by our cardiologist, according to the latest modified Brugada syndrome criteria [10]. There had been no events in his family history. A prophylactic implantable cardioverter-defibrillator was not applied because he had experienced no symptoms of Brugada syndrome. Preoperative transthoracic echocardiography did not reveal any abnormal findings.

We planned anesthesia with only sevoflurane and remifentanil, and postoperative intravenous patient-controlled analgesia (IV-PCA) with fentanyl to avoid local anesthetic-induced critical arrhythmias with regional anesthesia. Adhesive defibrillator pads were applied to the chest before induction of anesthesia. We used a 5-lead ECG including the right precordial leads positioned at V2 or V3 and continuously recorded ECG throughout the entire procedure. ST-segment elevation was analyzed at the J-point (J-point segment) and 60 ms from the J-point (ST-segment) in lead V2. Forced-air warming was applied to strictly control the body temperature between 36.5 and 36.8 °C during surgery because postoperative shivering can lead to fever-triggered ventricular arrhythmias. Isoproterenol was prepared for the occurrence of malignant arrhythmias.

**Fig. 2:**
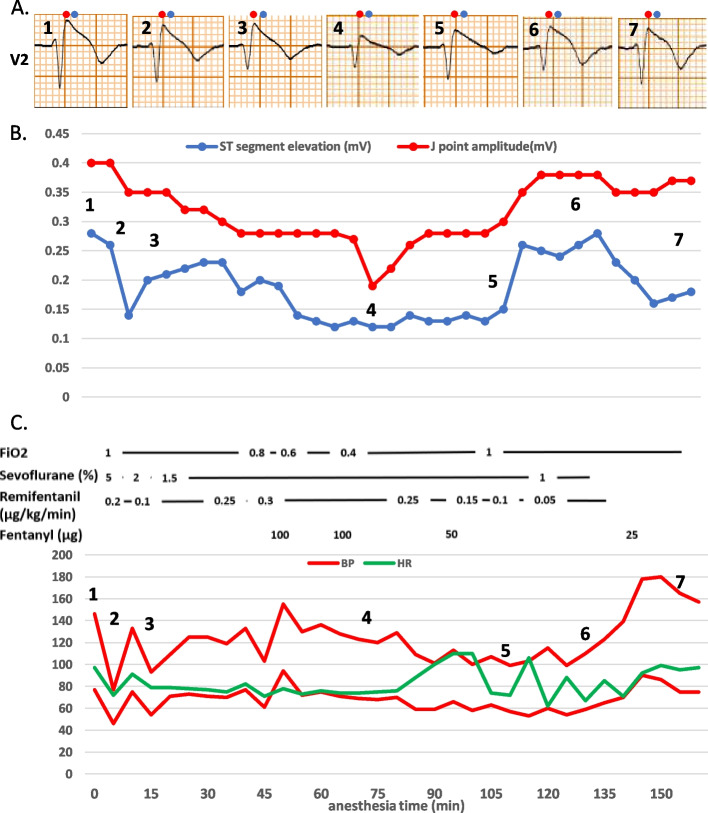
**A** Sequential electrocardiogram in lead V2 during anesthesia (red circle, J-point; blue circle, ST-segment). (1) Before induction of anesthesia. (2) After position adjustment of a double-lumen endotracheal tube. (3) After the postural change from supine to right lateral decubitus position. (4) After resection of the pleural mass. (5) At the end of surgery. (6) At eye-opening upon emergence from anesthesia. (7) Immediately before transfer to the high care unit. **B** Changes in the value of ST-segment (J-point) amplitudes in lead V2 in the operating room. **C** Anesthesia record for this case. BP, blood pressure; HR, heart rate

ECG immediately before induction showed coved type J-point and ST-segment elevation of 0.40 mV and 0.28 mV, respectively in V2 leads (Fig. 2A (1)). After a right radial arterial cannula was inserted, the patient underwent 5% sevoflurane inhalational induction with continuous intravenous infusion of remifentanil at 0.2 µg/kg/min. A tracheal intubation was performed with a double-lumen endotracheal tube after rocuronium 40 mg. Anesthesia was maintained with 1.0–2.0% sevoflurane, remifentanil at 0.05–0.30 µg/kg/min, and rocuronium at 10–20 mg/h. After position adjustment of the tube using fiberoptic bronchoscopy, we found a significant reduction in J-point and ST-segment elevation of 0.35 mV and 0.26 mV (Fig. 2A (2)), which was not affected by the postural change from supine to right lateral decubitus position (Fig. 2A (3)). VATS biopsy samples were taken from the parietal pleura. J-point and ST-segment elevations of 0.18–0.28 mV and 0.12–0.15 mV remained low during surgery (Fig. 2A (4, 5)). The surgery was completed without ventricular arrhythmia. After sevoflurane was discontinued and neuromuscular blockade was reversed with sugammadex, the trachea was extubated. During emergence from anesthesia, ECG changed with a gradual increase in J-point and ST-segment elevation, subsequently returning to the initial coved-type ST elevation (Fig. 2A (6, 7)). The patient showed no postoperative shivering or electrolyte anomalies.

The patient stayed in the high-care unit without any complications including critical arrhythmia. He was transferred to a general ward on the third postoperative day.

## Discussion

We anesthesiologists need to be aware of the potential proarrhythmic effects and interactions of drugs used during the perioperative period for patients with Brugada syndrome. While propofol is one of the most widely used intravenous anesthetics, there has been extensive debate regarding the safety of propofol bolus or continuous infusion in patients with Brugada syndrome because of its arrhythmogenic effects [6]. Recently, Flamée et al. reported its safe use without critical arrhythmias in the operating theater or the intensive care unit [7]. In addition, they also reported that induction with propofol or etomidate did not affect ST-segment elevation [9] whereas Ciconte et al. showed that propofol significantly reduced ST-segment elevation immediately after induction [8]. Sevoflurane, which also should be applied with caution because of its QT-prolonging effects, has been used without complications in patients with Brugada syndrome under ECG monitoring [6]. A previous report showed that sevoflurane (2–3%)-based anesthesia following a propofol bolus reduced ST-segment elevation 20 min after induction [8]. However, the effects of such anesthetics on ST-segment elevation in patients with Brugada syndrome throughout general anesthesia have not been elucidated. Based on these previous reports, we have developed an anesthetic plan with fewer agents whenever possible. Although propofol can likely be used safely during induction, we considered its arrhythmogenic effects and increased risk of propofol infusion syndrome [6]. Our plan can realize induction and maintenance of anesthesia with sevoflurane alone. In the present case, we monitored ST-segment morphology in a patient with the Brugada type 1 pattern and revealed sustained mitigation of ST-segment elevation throughout sevoflurane and remifentanil infusion. Therefore, this present case suggests that safe anesthetic management of patients with Brugada syndrome may depend on whether the anesthetics used can reduce ST-segment elevation.

The mechanism by which sevoflurane reduces ST-segment elevation has not been well investigated. Brugada syndrome mutation in KCNE3 (MiRP2) has been suggested to result in increased transient outward K^+^ current (Ito) and Ito channel densities in the right ventricular epicardium [11, 12]. In combination with a reduction in depolarizing current due to the concomitant presence of an SCN5A loss-of-function mutation, this causes loss of action potential dome in the epicardium resulting in ST-segment elevation and consequently phase-2 re-entry upon arrival of a subendocardial action potential wavefront causing VF [3, 13]. In a recent study [14] using cloned human cardiac K^+^ channels, sevoflurane inhibited Kv4.3 cardiac K^+^ channel currents, suggesting inhibition of Ito channels. Moreover, a high density of Ito channels is implicated in the enlargement of the epicardial arrhythmogenic substrate, whose marked reduction was observed during sevoflurane-based anesthesia with changes in ST-segment elevation [8]. We consider that the inhibiting effects of sevoflurane on Ito channels may be involved in the outcome of the present case.

There are many other risk factors for worsened ST-segment elevation and VT/VF including hyperthermia, electrolyte anomalies, vasopressor drug use, parasympathetic stimulation, and local anesthetics [6]. Although opioids are considered relatively safe to use, high doses of remifentanil should be used with caution because it increases parasympathetic tone. Fentanyl is preferred to control severe postoperative pain that could trigger a vagal reflex. We hesitated to use epidural or peripheral nerve blocks because all local anesthetics are sodium channel blockers that would induce arrhythmias. In the present case, the VATS procedure was relatively noninvasive, and IV-PCA with fentanyl provided sufficient analgesia without malignant arrhythmia in the perioperative period.

In summary, we report a case of a patient with Brugada type 1 pattern undergoing VATS procedure in whom sustained mitigation of ST-segment elevation throughout anesthesia with only sevoflurane and remifentanil. Monitoring ST segments in the right thoracic lead throughout general anesthesia may be useful to determine the risk of transition to ventricular arrhythmias with current anesthesia methods in patients with Brugada syndrome.

## Data Availability

Please contact the author for data requests.

## Abbreviations

ECG: Electrocardiogram
VT/VF: Ventricular tachycardia/fibrillation
VATS: Video-assisted thoracoscopy surgery
IV-PCA: Intravenous patient-controlled analgesia
